# Soft shape-programmable surfaces by fast electromagnetic actuation of liquid metal networks

**DOI:** 10.1038/s41467-022-31092-y

**Published:** 2022-09-23

**Authors:** Xinchen Ni, Haiwen Luan, Jin-Tae Kim, Sam I. Rogge, Yun Bai, Jean Won Kwak, Shangliangzi Liu, Da Som Yang, Shuo Li, Shupeng Li, Zhengwei Li, Yamin Zhang, Changsheng Wu, Xiaoyue Ni, Yonggang Huang, Heling Wang, John A. Rogers

**Affiliations:** 1 Querrey Simpson Institute for Bioelectronics, Northwestern University, Evanston, IL USA; 2 Department of Materials Science and Engineering, Northwestern University, Evanston, IL USA; 3 Department of Mechanical Engineering and Materials Science, Duke University, Durham, NC USA; 4 Department of Mechanical Engineering, Northwestern University, Evanston, IL USA; 5 Department of Biostatistics and Bioinformatics, Duke University, Durham, NC USA; 6 Department of Civil and Environmental Engineering, Northwestern University, Evanston, IL USA; 7 Department of Biomedical Engineering, Northwestern University, Evanston, IL USA; 8 Department of Neurological Surgery, Northwestern University, Evanston, IL USA; 9 Department of Electrical and Computer Engineering, Northwestern University, Evanston, IL USA; 10 Department of Chemistry, Northwestern University, Evanston, IL USA

**Keywords:** Mechanical engineering, Polymers, Displays, Actuators, Metamaterials

## Abstract

Low modulus materials that can shape-morph into different three-dimensional (3D) configurations in response to external stimuli have wide-ranging applications in flexible/stretchable electronics, surgical instruments, soft machines and soft robotics. This paper reports a shape-programmable system that exploits liquid metal microfluidic networks embedded in an elastomer matrix, with electromagnetic forms of actuation, to achieve a unique set of properties. Specifically, this materials structure is capable of fast, continuous morphing into a diverse set of continuous, complex 3D surfaces starting from a two-dimensional (2D) planar configuration, with fully reversible operation. Computational, multi-physics modeling methods and advanced 3D imaging techniques enable rapid, real-time transformations between target shapes. The liquid-solid phase transition of the liquid metal allows for shape fixation and reprogramming on demand. An unusual vibration insensitive, dynamic 3D display screen serves as an application example of this type of morphable surface.

## Abstract (web summary)

Low modulus materials that can change shape in response to external stimuli are promising for a wide range of applications. The authors here introduce a shape-reprogrammable construct, based on liquid metal microfluidic networks and electromagnetic actuation, that supports a unique collection of capabilities.

## Introduction

Materials that can change in shape in a reversible and programmable manner are of rapidly growing interest for use in deployable structures^1,2^, robotic systems^3,4^, and biomedical devices^5–9^. Among the many different classes of such platforms, sheets/membranes that can actively transform into different 3D configurations upon a programmable, external stimulus are particularly notable for their applications in tunable optics^10,11^, soft machines^12^, biomimetic skins^13^, and structures for aerodynamic drag control^14,15^. A variety of materials can be exploited to affect such types of geometrical transformations, including most prominently those that rely on properties of liquid crystal elastomers^16–18^, shape memory polymers and alloys^19,20^, responsive hydrogels^21–23^, conductive polymers^24^, dielectric elastomers^25,26^, metal nanowire networks^27,28^, and carbon nanotubes and graphene^29,30^. Other schemes rely on active control over processes in compressive buckling^31–33^ or folding deformations^8,34–39^. Most of these approaches, however, suffer from one or more of the following limitations: (i) use of discrete mesh/lattice structures to approximate smooth surfaces^40–42^, (ii) inability to reversibly transform into multiple shapes^23,43^, (iii) modest range of accessible 3D geometries from a single design^44,45^, (iv) power inefficiencies in operation^13,23^, and (v) relatively slow speeds for shape morphing^16,46,47^. This paper adapts recently reported concepts in soft actuators^48^ as the basis for a fast, programmable, shape-morphing materials system that uses cross-bar arrays of liquid metal traces embedded in sealed, elastomeric microfluidic structures. This platform supports on-demand, real-time reversible transformations of functional membranes into diverse varieties of complex, continuous 3D surfaces, with capabilities in rapid, continuous switching between sequences of geometries. Specifically, shapes of these liquid metal/elastomer hybrid materials can be precisely controlled by varying the distribution of currents that pass through a collection of liquid metal ribbon structures while in the presence of a static magnetic field. Distributed Lorentz forces produced in this manner simultaneously act on different regions of the surface, balanced by restoring forces associated with the deformed elastomer, to determine the resulting shape. This system offers rapid, reversible shape morphing responses and continuous programmability, with advantages in speed and power consumption compared to existing mechanisms that use thermal^49–51^, chemical^52,53^, or mechanical stimuli^31,54,55^. Furthermore, despite the requirement of a magnetic field, this purely electronic system avoids the complexity, limited reliability and constrained scalability associated with conventional pneumatically actuated systems^13^ that use pumps and valves. Additional features distinguish this system from a recently reported analog that utilizes an open mesh of serpentine-shaped filamentary solid metal traces^56^, such as (i) smooth, continuous surfaces with shapes that can be described quantitatively using non-linear elastic membrane mechanics theory, validated by high resolution 3D digital image correlation^57^ (3D-DIC) techniques, (ii) large-scale shape libraries for rapid programming, and (iii) capabilities in shape fixation and reprogramming between different geometries that can support mechanical loads. An unusual 3D dynamic display system provides a representative example of an application of these morphable materials in the area of advanced optics.

## Results and discussion

### Concepts and design principles

**Fig. 1:**
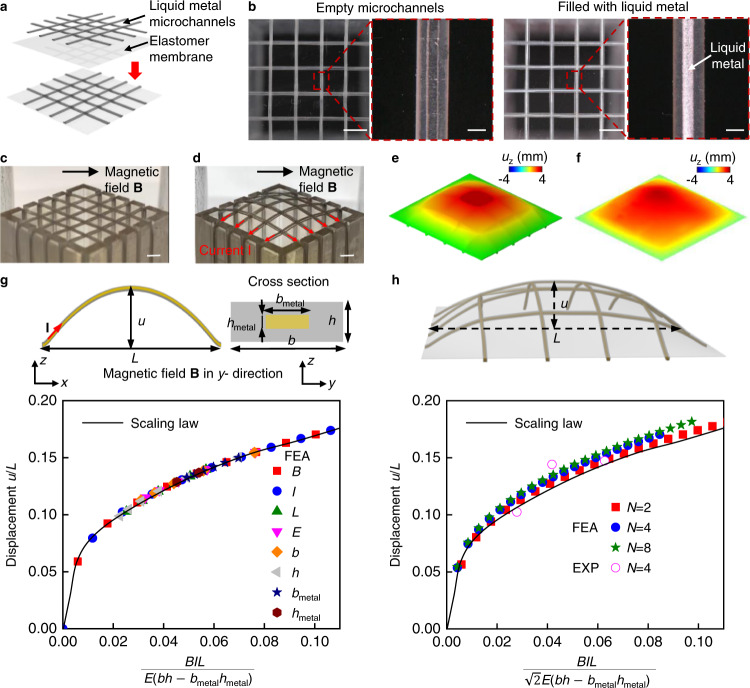
Programmable surfaces enabled by electromagnetic actuation of liquid metal networks. **a** Schematic illustration of the fabrication process. Soft microfluidic channels filled with liquid metal serve as conducting ribbons bonded onto a thin elastomeric membrane (~5 µm) to form the programmable surface. **b** Optical image of the programmable surface before and after filling with liquid metal. The elastomeric membrane is optically transparent in regions not occupied by liquid metal. Scale bar, 5 mm for the surface, 500 µm for the exploded view illustration of the microchannels. Optical images of the surface in the initial flat configuration (**c**) and at maximum deformation (**d**). Various 3D shapes can be obtained through the action of Lorentz forces by controlling the electric currents passing through each liquid metal ribbon. Scale bar, 5 mm. FEA (**e**) and 3D Digital Image Correlation (3D-DIC; **f**) results of the surface in **d**. **g** Scaling law results for the maximum deformation of an isolated liquid metal ribbon as a function of ribbon geometries, material properties, magnetic field strength, and applied current. **h** Plots of the maximum surface deformation as a function of the scaling law parameters with different ribbon numbers (2-by-2, 4-by-4, and 8-by-8).

Figure 1 presents a schematic illustration of the materials and methods for actuation. The particular soft microfluidics structure shown here incorporates thin layers (~100 µm) of a low modulus (*E* = ~232 kPa) formulation of a silicone elastomer (Dragon Skin 10, Smooth-On), molded and assembled using established approaches in soft lithography. A laser cutter (LPKF ProtoLaser R) defines isolated straight ribbons (width *b* = 900 µm) that contain the microfluidic channels (cross section: width *b*_metal_ = 300 µm, height *h*_metal_ = 200 µm). An injection process fills these channels with eutectic gallium-indium (EGaIn; 75% Gallium, 25% Indium by weight) as a liquid metal conducting ribbon. The total thickness *h* of each of these ribbons is ~400 µm including the encapsulated liquid metal. Assembling these ribbons into a grid pattern and bonding them onto a thin (*h*_membrane_ = 5 µm) layer of a different type of silicone elastomer (Ecoflex 00—20: Silicone Thinner = 10:8 by weight, Smooth-On; *E*_membrane_ = 6.8 kPa) completes the fabrication, as in Fig. 1a and b. Details are in the Methods section and Supplementary Figs. 1 and 2. The resulting membrane is optically transparent in regions not occupied by the liquid metal and it is extremely compliant (tensile rigidity *E*_membrane_*h*_membrane_*L*_s_ = ~1.3 mN per unit strain, where *L*_s_ = *~*39 mm is edge length of the surface) due to its small thickness and low effective elastic modulus. The membrane deforms ~10% under the weight of a 0.01 g water droplet (Supplementary Fig. 3). Other choices in dimensions, geometries, and constituent materials are possible.

When placed in a magnetic field, this surface (initially in flat, 2D geometry; Fig. 1c) can be programmed to transform into various 3D shapes through the action of Lorentz forces generated by passing electric currents (**I**) through the liquid metal ribbons (Fig. 1d). Specifically, each ribbon serves as an independent control channel that drives local deformations in the membrane. An external power source defines the magnitude (*I*) and the direction of the current in each ribbon, via the use of a microcontroller (Arduino Mega 2560). This arrangement enables precise control of both the magnitude and direction of the Lorentz forces (**F** *= L* **I** × **B**) that act on each ribbon, where **B** is the magnetic field and *L* is the ribbon length. Most experiments reported here use a non-uniform magnetic field generated by a single permanent cuboidal block magnet (38.1 mm × 38.1 mm × 38.1 mm) placed ~5 mm below the surface (39 mm × 39 mm). The field in this case is mostly parallel to the surface plane, with an amplitude of ~0.2 T at the center and gradually decreasing to ~0.1 T at the edges. The ability to achieve shape-morphing on demand in non-uniform magnetic fields greatly enhances the practicality of the system. Some experiments rely on a uniform magnetic field created by placing two permanent disk magnets (diameter = 76.2 mm, thickness = 12.7 mm) in parallel to each other separated at a distance of 45 mm, with the programmable surface (25 mm × 25 mm) placed in between. Here, the magnetic field is parallel to the surface plane, oriented at a 45° angle with respect to the ribbons, with a uniform magnitude of ~0.15 T. Details appear in Supplementary Fig. 4. For the examples reported here, the current that passes through each channel has a magnitude between 0 and ±0.5A. With a maximum magnetic field *B* ~ 0.2 T and a maximum ribbon length *L* *~* 48 mm, the Lorentz force per channel ranges from 0 to 4.8 mN, in both the upward and downward directions. With a resistance for each channel *R* *~* 0.5 Ω, the maximum power consumption per ribbon is 125 mW, calculated via *P* = *I*^*2*^*R*.

3D-DIC methods define full-field displacements and strains across the entire area of the system during operation. Figure 1d–f presents predictions of the surface at maximum deformation determined by finite element analysis (FEA) along with corresponding experimental measurements. An optical image captured using a single camera (Fig. 1d) indicates a shape profile that is similar to the FEA result (Fig. 1e). Quantitative imaging by 3D-DIC (Fig. 1f) also agrees with FEA, as shown in the color maps. The essential physics can also be captured using theoretical methods. For an isolated ribbon structure with two fixed ends, the effect of bending becomes negligible when the out-of-plane deformation is large (e.g., maximum deformation *u*/*L~*10%) such that in-plane tension dominates. In this condition, dimensional analysis suggests that *u*/*L* increases with the total force *BIL* divided by the in-plane tensile rigidity *E*(*bh−b*_metal_*h*_metal_) according to the following scaling law,

1 $$\frac{u}{L}=G\left[\frac{{BIL}}{E\left({bh}-{b}_{{{{{{\rm{metal}}}}}}}{h}_{{{{{{\rm{metal}}}}}}}\right)}\right]$$

where *G* is a nonlinear function determined by FEA. Equation (1) matches with FEA results (Fig. 1g), thereby validating the use of computational techniques in the design of these types of programmable surfaces. Due to the extremely small thickness (~5 µm) and low modulus (~6.8 kPa) of the membrane, the tensile rigidity of the membrane is ~2 orders of magnitude lower than that of an isolated ribbon. As a result, the maximum deformation of the programmable surface with an *N*-by-*N* array of ribbons can also be well predicted by Eq. (1) with an error less than 10%. Figure 1h shows the maximum deformation of the programmable surface with different number of ribbons as a function of the dimensionless parameter from the scaling law. Since both the total force *2NBIL* and the total tensile rigidity *2NE*(*bh−b*_metal_ *h*_metal_) are linearly proportional to the total number of ribbons 2*N*, increasing the number of ribbons has little effect on the maximum out-of-plane deformation of the surface. This invariance of maximum deformation on the total number of ribbons supports a simple scaling process for enhancing the shape fidelity without significantly altering the programming control. A 4-by-4 design represents the focus of studies described in the following sections.

### Fast, diverse 3D shape morphing and reprogramming

Transformations from the initial planar configuration can occur rapidly into a diverse collection of 3D shapes. Even a relatively simple 4-by-4 ribbon design (8 ribbons in total) provides close to half a million possible shapes (5^8^ = ~0.4 million) when implemented with only five levels of control over the currents supplied to each ribbon. Transformations between these shapes are fast due to the electromagnetic actuation mechanism. For an isolated ribbon, FEA predicts the time to reach a stable deformation is ~50 ms when accounting for dynamic effects (see Supplementary Fig. 5 and Methods), which agrees well with experimental measurement of ~30 ms. Supplementary Table 1 presents a summary of the various shape morphing systems and their response time^58–60^. The system presented in this work is among the fastest. For the entire surface, the experimentally measured total response time for developing a full shape starting from a flat configuration is ~300 ms, dominated by the viscoelastic response of the membrane (~250 ms for the membrane to reach maximum deformation). This result is also in agreement with FEA prediction, as shown in Supplementary Fig. 5. The switching speed between two arbitrary shapes is the sum of the time to develop the first shape (~300 ms), the time to process the script for the next shape (~50 ms), followed by the time to develop the second shape (~300 ms). Supplementary Movie 1 illustrates these processes in slow motion with timestamps.

**Fig. 2:**
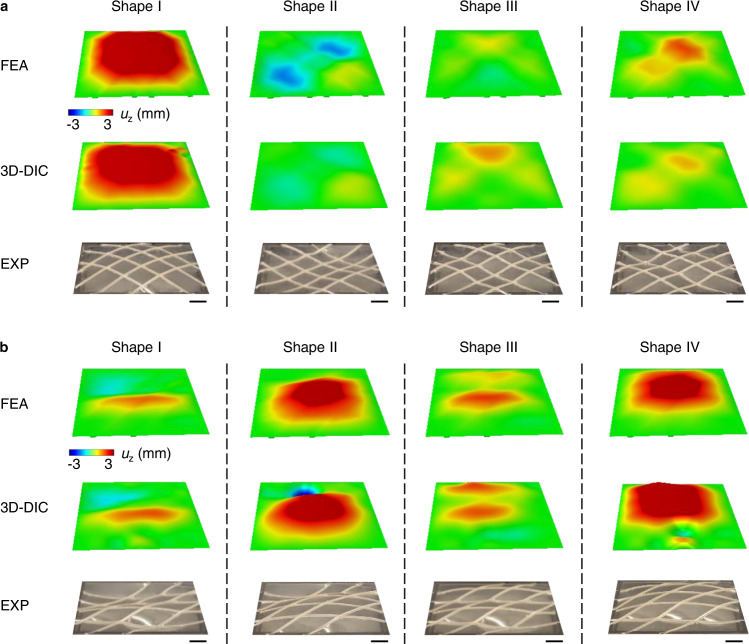
3D shapes transformed from the programmable surface. **a** FEA predictions and experimental results (3D-DIC and optical images) of four representative 3D shapes transformed from the programmable surface featuring an orthogonal and symmetric ribbon layout (45°/−45°) in a non-uniform magnetic field associated with a single permanent block magnet. **b** FEA predictions and experimental results (3D DIC and optical images) of four representative 3D shapes transformed from the programmable surface featuring a non-orthogonal and asymmetric ribbon layout (0°/45°) in the same magnetic field as in **a**. Scale bars, 5 mm.

These transformations are fully reversible (i.e., shape reprogramming) due to the nature of the constituent materials and the mechanisms for actuation. Figure 2a presents four representative 3D shapes, each transformed from a 2D flat sheet that uses a collection of ribbons in an orthogonal lattice design (45°/−45°) actuated in a nonuniform magnetic field associated with a single permanent block magnet. Each case shows good agreement between FEA and experimental results (optical and 3D-DIC), even under these nonuniform magnetic field conditions. The difference between FEA and 3D-DIC measurements is typically less than 2% (normalized by the edge length of surface *L*_*s*_) for ~90% of the measured points (Supplementary Fig. 6). Detailed analysis of the errors associated with the intrinsic uncertainties of 3D-DIC reconstruction appears in Methods and Supplementary Fig. 7. Additional results for shape transformations in a uniform magnetic field are available in Supplementary Fig. 8. FEA predicts that the maximum principal strain of ~6% occurs around the edges in Shape I (Fig. 2a; Supplementary Fig. 9). Supplementary Movie 2 presents 20 shapes, including those in Fig. 2a, and illustrates the fast and fully reversible nature of these transformations. Tracking five representative points on the surface (Supplementary Fig. 10) as a function of time serves as the basis for quantifying the reversibility during a process of programmed change between 50 consecutive shapes, returning to the initial 2D planar configuration between each transformation. As shown in Supplementary Fig. 10, the displacements of these points return to zero after each cycle, consistent with the reversible operation. Tracking the displacement response of the center point on the surface using 3D-DIC during a loading and unloading cycle where currents increase from 0 to 0.5 A then decrease back to 0 A suggests negligible hysteresis in the system (Supplementary Fig. 11). Programmable surfaces that use non-orthogonal and asymmetric ribbon layouts are also possible, as shown in the examples of Fig. 2b for a 0°/45° ribbon layout. Supplementary Movie 3 presents results of fast switching between 20 shapes achieved with this system. Supplementary Figs. 12 and 13 show the underlying deformations of the liquid metal ribbons and the applied currents that correspond to the various 3D continuous shapes shown in Fig. 2.

### 3D DIC-enabled rapid transforming to target shapes

**Fig. 3:**
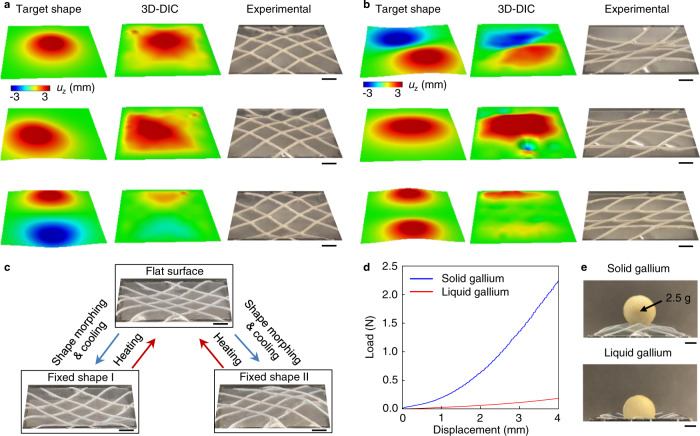
Target shape programming and shape fixation. Various target shapes realized by the programmable surfaces with 45°/−45° (**a**) and 0°/45° (**b**) ribbon layouts, respectively. **c** Shape fixation and reprogramming utilizing liquid metal phase transition. **d** Experimental results of the different force–displacement response of a surface that incorporates a 4-by-4 array of gallium ribbons in solid and liquid states. **e** Load bearing capability of the surface with gallium ribbons in solid and liquid states. Scale bars, 5 mm.

A simulation-guided approach can be used to achieve targeted shapes, but with practical limitations associated with computational time, typically on the order of hours for one shape prediction using a workstation with 40-core, 2.40 GHz CPU, and 64 GB memory. An alternative, empirical scheme relies on building a library database by fast switching between a broad, diverse sequence of shapes while performing high-speed 3D imaging. The results yield relationships between large numbers of shapes and the corresponding currents in the ribbons. Within a few hours, data of this type can be acquired for ~7000 shapes. (An FEA approach using the computational resource described above would require ~2 years of computational time.) Details appear in Supplementary Figs. 14 and 15. Target shapes defined in Supplementary Note 1 and Supplementary Tables 2 and 3 can be matched to a selection within this library using a cost function defined by the sum of squares differences between the vertical displacements of a collection of locations across the surface. This matching process can be achieved within 1 ms using the same workstation for FEA predictions. Figure 3a and b shows some examples of target shapes realized using programmable surfaces with 45°/−45° and 0°/45° ribbon layouts respectively. Examples include a dome shape with four relatively flat corners (Fig. 3a, top), and wave shapes with long (one peak and one valley across the sample width; Fig. 3b top) and short (two peaks and one valley across the sample width; Fig. 3b bottom) wavelengths. The surface can be programmed to switch between any collections of these shapes rapidly and on demand, as shown in Supplementary Movies 4 and 5.

### Shape fixation

The liquid–solid phase transition associated with the liquid metal features can be exploited for shape fixation. Here, a materials system of pure gallium (melting temperature: 29 °C) and silicone microfluidic channels with circular cross-sections (ID = 0.30 mm, OD = 0.63 mm, *E* = 3.25 MPa, McMaster-Carr) serves to demonstrate this feature at room temperature (~20 °C). Due to the combined effects of Joule heating and supercooling^61^, gallium remains in the liquid state even at room temperature (~9 °C below its melting point), allowing for the same, versatile shape transformations demonstrated previously using EGaIn (melting temperature: 17 °C). Carefully spraying a refrigerant (1,1,1,2-Tetrafluoroethane, Fisher Scientific) onto the surface decreases the gallium temperature significantly below its melting point to ~−50 °C, via effects of evaporative cooling. Performing this process while maintaining the desired current distribution transforms the gallium to its solid state in less than 1 s and consequently fixes the shape. The surface maintains this shape without external currents or magnetic field, provided that the temperature remains below 29 °C (Fig. 3c, bottom) which includes room temperature (~20 °C). For shape-reprogramming, Joule heating that follows from passing current through the gallium (0.5 A applied at room temperature melts the gallium within 30 s; Supplementary Fig. 16) or from other mechanisms (e.g., IR illumination) liquefies the solid gallium ribbons, thereby returning the surface to its initial flat configuration and allowing for subsequent shape transformations (Fig. 3c, top and Supplementary Movie 6). This shape fixation and reprograming scheme can be applied repeatedly when the ambient temperature remains below 29 °C. For applications where the ambient temperature is higher than 29 °C, other metal/alloy choices can be considered. The force–displacement curves in Fig. 3d show that the stiffness of a surface that incorporates a 4-by-4 array of ribbons with gallium in the liquid state is approximately ~10× lower. The stiffness difference at various levels of vertical compressive displacement appears in Supplementary Fig. 17. The stiffness increase due to the liquid to solid phase transition of gallium enables the load bearing capability of the structure. Figure 3e and Supplementary Movie 7 show support of a ball with a mass of ~2.5 g. Additional FEA results in Supplementary Fig. 18 show that the surface (*h*_membrane_ = 100 μm and *E*_membrane_ = 100 kPa) deforms ~1.5 mm under 1 kPa of pressure applied over a circular area with diameter *D* = 20 mm at the center. The deformation decreases to ~0.5 mm when the total number of ribbons are increased from 4-by-4 to 16-by-16. The change in stiffness enabled by the liquid to solid phase transition of liquid metal is one of the many techniques to achieve “variable stiffness” on demand^62^. Other notable approaches utilize shape memory materials^63^ and electroactive polymers^64^, jamming^65^ or buckling^66^ mechanisms, as well as electro-bonded laminates^67^, multi-layered beams^68^, and aerofoils^69^ in aerospace structures.

### 4D programmability

**Fig. 4:**
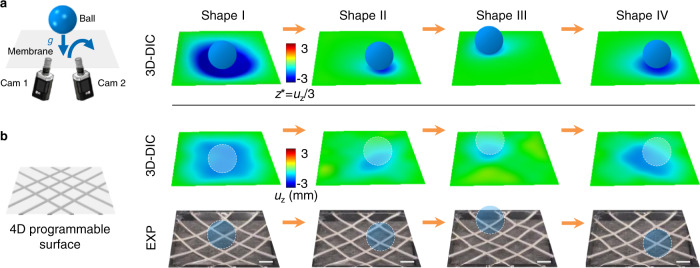
4D programmability. **a** A dynamic shape changing sequence associated with dropping a ball onto a test membrane under the action of gravity, as captured by 3D-DIC. **b** Experimental results (3D-DIC and optical images) of the programmable surface reproducing the dynamic physical process in **a**, but without the ball. Scale bars, 5 mm.

The unique set of capabilities described above provide the basis for a 4D programmable material—i.e., a system capable of imitating dynamic, sequential shape-morphing processes, as opposed to discrete geometries. A simple demonstration involves reproducing the dynamic physical process associated with a sphere dropping onto an elastomeric membrane under the action of gravity, as captured by high-speed 3D imaging and then subsequently reproduced by shape-programming, as presented in Fig. 4a. Here, as a test membrane undergoes continuous shape changes caused by movement of the ball, imaging captures the precise 3D coordinates associated with a collection of time-evolving shapes in a sequence. This sequence defines target shapes for the programmable surface. Using the fast inverse-design scheme described previously, a temporal sequence of applied currents can be determined within seconds. Applying these time dependent currents yields a moving surface that reproduces the dynamics of the test membrane with the ball, but without the ball, as shown in Fig. 4b and Supplementary Movie 8.

### Vibration insensitive projection screens and 3D display systems

**Fig. 5:**
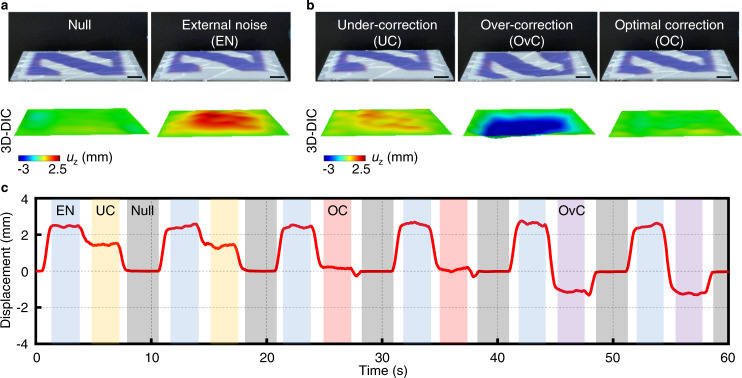
Noise canceling capability. **a** Optical (top) and 3D-DIC images (bottom) of the programmable surface with the letter “N” projected onto it (left) and bulging upwards due to external vibrational noise (right). **b** Optical (top) and 3D-DIC images (bottom) of the programmable surface actuated with different current intensities to find the optima to cancel the noise effect. **c** The maximum displacement on the surface as a function of time at different phases of noise cancellation. Scale bars, 5 mm.

This sort of dynamic, programmable operation creates opportunities in various applications, including those that involve optics and information display. For example, doping the silicone used in these systems yields a platform that can serve as a dynamically programmable projection screen. As an adaptive optical element, appropriately programmed changes in shape can reduce/cancel parasitic motions induced by vibrations or other mechanical disturbances. Figure 5 illustrates this concept with the projection of a purple letter “N” (flat state, Fig. 5a, left). Introducing vibrational noise causes the screen to bulge upwards (Fig. 5a, right) such that the top portion of the letter “N” becomes invisible. Optimized actuation of the surface eliminates this bulge to recover a planar geometry (Fig. 5b, right). Insufficient current amplitudes lead to under-correction, where the top portion of the letter “N” remains invisible (Fig. 5b, left). Excessive currents cause over-correction, such that the lower and middle portion of the letter moves out of sight (Fig. 5b, middle). 3D-DIC captures this noise canceling process in real-time (Fig. 5a and b, bottom row). This process also appears as Supplementary Movie 9. Figure 5c presents the displacement of the center point on the surface as a function of time measured by 3D imaging quantitatively, demonstrating the active noise canceling capability.

**Fig. 6:**
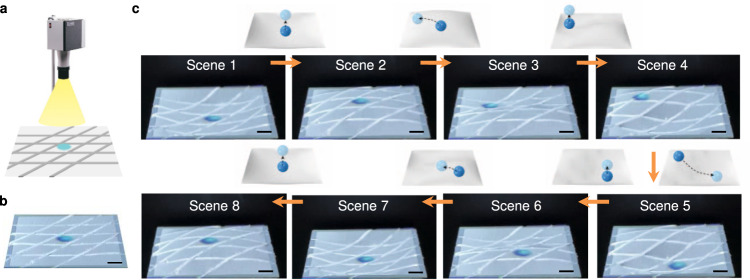
Applications of the 4D programmable surface as an unusual type of dynamic 3D display system. Schematic illustration (**a**) and optical image (**b**) of the programmable surface serving as a projection screen. **c** Schematic illustrations and selected frames from Supplementary Movie 10 of a moving ball projected onto the surface as the shape of the surface morphs to create an illusion of actual physical interactions due to surface modulation, gravity, and inertial effects. Scale bars, 5 mm.

A compelling extension of this concept is in a class of dynamic 3D display system that coordinates morphing of the geometry of the screen with moving images projected onto it. Figure 6a and b presents a schematic illustration (Fig. 6a) and an optical image (Fig. 6b) of a setup where such changes in shape create interesting effects. Figure 6c presents an illustration in which a video of a moving ball projects onto the surface as the shape of the surface morphs to create an illusion of physical interactions. Specifically, the projected ball appears to engage with the surface in a manner similar to an actual ball moving and modulating the shape of the surface due to gravity and inertial effects. In this example, the ball lifts from the center, slides into one of the four corners, lifts again, slides across the surface diagonally into the opposite corner and finally slides back to the starting center position. This dynamic 3D movie appears in Supplementary Movie 10. The ability to morph a traditional 2D display into a dynamic 3D surface may complement or extend holographic display concepts, with potential use in virtual or augmented reality systems.

In summary, the concepts introduced here establish a versatile and robust design and fabrication scheme for shape-programmable surfaces. The approach utilizes cross-bar arrays of liquid metal soft microfluidic structures and electromagnetic actuation, yielding a set of capabilities previously unattainable in a single materials system. Key features include fast and continuous surface shape morphing and reprogramming with access to a diverse set of 3D shapes originating from a single 2D planar configuration and well-controlled 4D electronic programmability. Computational methods capable of predicting complex 3D shape transformations in non-uniform magnetic fields in conjunction with advanced 3D imaging techniques that enable fast and precise quantification of surface deformation serve as both design and characterization tools. A 3D display system with noise cancellation capability represents one example of many in applied optics, where the 3D movement of the surface is time synchronized with projected video content. These and other unusual features of this simple materials system suggest a broad range of opportunities in flexible electronics, soft robotics, and biomedical devices.

## Methods

### Fabrication of liquid metal ribbons

Preparation of liquid metal ribbons began with 3D printing (printer: Form 3, Formlabs, material: Clear V4) to form a mold with patterns in the geometries of the microfluidic channels (cross section width = 300 µm, height = 200 µm). Spin coating a layer of silicone elastomer (Dragon Skin 10, Smooth-On) at 1000 rpm for 30 s onto the mold and curing at 75 °C for 30 min yielded the channel layer (thickness = ~100 µm). Spin coating another layer of Dragon Skin at 3000 rpm for 30 s and curing at 75 °C for 30 min formed the capping layer (thickness = ~100 µm). Both layers were treated with corona discharge for 2 min to facilitate bonding between them. Baking on a hotplate at 75 °C for 30 min with some applied pressure ensured strong adhesion. A laser cutting (ProtoLaser R, LPKF Laser & Electronics AG) process defined the geometric outlines of ribbons (cross section width = 900 µm, thickness = ~400 µm) containing the microfluidic channels (cross section width = 300 µm, height = 200 µm). Injecting liquid metal into the microfluidic channels completed the fabrication of the liquid metal ribbon structures.

### Fabrication of freestanding ultrathin elastomeric membrane

Preparation of a freestanding elastomeric membrane began with mixing soft silicone elastomer (Ecoflex 00—20, Smooth-On) with silicone thinner (Silicone Thinner, Smooth-On) at a 10:8 weight ratio (silicone: thinner). A thin layer of polyvinyl alcohol (PVA) solution (20 wt%) was spin coated (3000 rpm for 30 s) and cured (110 °C on a hotplate for 1 min) on a clean glass slide (75 mm × 50 mm) serving as a sacrificial layer. The diluted silicone mixture was then spin cast (6000 rpm for 30 s) onto the PVA layer and cured (75 °C in an oven for 30 min), to yield a thin elastomeric membrane (thickness = ~5 µm). A 3D printed frame (thickness = 8 mm, printer: Form 3, Formlabs, material: Flexible 80A) was bonded onto this membrane using a silicone adhesive (Kwik-Sil, World Precision Instruments). Immersing the entire assembly in water overnight dissolved the underlying PVA, to release the membrane from the glass side with the frame to facilitate handling.

### Finite element analysis

The computational modeling used commercial FEA software Abaqus in conjunction with a homemade Python script. The model predicted the deformation of the liquid metal ribbons and the programmable surface under electromagnetic actuation. The electric module in Abaqus simulated the distribution of current densities with the applied voltages as the boundary conditions. The Python script then calculated the distribution of the Lorentz forces and transferred the information to the mechanics module in Abaqus as body forces to predict the deformation. The simulation was divided into several loading steps such that a small portion (<5%) of total electric current was added in each step. The nonuniform magnetic field generated by the cuboidal magnet (length 2*a*, magnetization *M*) was calculated by the formula below^70^ (details appear in Supplementary Fig. 4)

$${B}_{x} 	=\frac{{\mu }_{0}M}{4\pi }\,{{{{\mathrm{ln}}}}}\left[\frac{{F}_{2}(-x,z,-y){F}_{2}(x,z,y)}{{F}_{2}(x,z,-y){F}_{2}(-x,z,y)}\right]\\ {B}_{y} 	=-\frac{{\mu }_{0}M}{4\pi }\,{{{{\mathrm{ln}}}}}\left[\begin{array}{c}{F}_{1}(-z,x,y)+{F}_{1}(-z,x,-y)+{F}_{1}(-z,-x,y)+{F}_{1}(-z,-x,-y)\,+\\ {F}_{1}(z,x,y)+{F}_{1}(z,x,-y)+{F}_{1}(z,-x,y)+{F}_{1}(z,-x,-y)\end{array}\right]\\ {B}_{z} 	=\frac{{\mu }_{0}M}{4\pi }\,{{{{\mathrm{ln}}}}}\left[\frac{{F}_{2}(-z,x,-y){F}_{2}(z,x,y)}{{F}_{2}(z,x,-y){F}_{2}(-z,x,y)}\right],$$

with

$${F}_{1}(x,y,z) 	=\arctan \left[\frac{(x+a)(y+a)}{(z+a)\sqrt{{(x+a)}^{2}+{(y+a)}^{2}+{(z+a)}^{2}}}\right]\\ {F}_{2}(x,y,z) 	=\arctan \left[\frac{\sqrt{{(x+a)}^{2}+{(y-a)}^{2}+{(z+a)}^{2}}+a-y}{\sqrt{{(x+a)}^{2}+{(y+a)}^{2}+{(z+a)}^{2}}-a-y}\right],$$

and where *μ*_0_ is the magnetic permeability of free space. The magnetization was *M* = 1.1 × 10^6^ A/m (*μ*_0_ *M* = 1.38 T), measured experimentally using a Gauss meter (PCE-MFM 3000, PCE instruments). The thin membrane was modeled by four-node shell elements. The walls of the microfluidic channels were modeled by eight-node solid elements. As the liquid metal was fully sealed inside microfluidics channels, no flow would occur. The liquid metal was modeled as an incompressible solid with negligible rigidity using eight-node solid elements. A refined mesh with feature sizes smaller than 1/5 of the thickness of the channel wall was adopted to ensure accuracy. The Mooney-Rivlin hyperelastic constitutive model was adopted by all materials, with the elastic modulus (*E*) and Poisson’s ratio (*ν*) being *E*_membrane_ = 7.0 kPa and *ν*_membrane_ = 0.49 for the membrane, *E* = 232 kPa and *ν* = 0.49 for the channel walls, *E*_liquid_ = 1.0 kPa and *ν*_liquid_ = 0.49 for the liquid metal (EGaIn and gallium), and *E*_solid_ = 9.8 GPa and *ν*_solid_ = 0.47 for the solid gallium. The dynamic response in Supplementary Fig. 5 was simulated by the implicit dynamic module in Abaqus. A stiffness proportional damping factor *β* = 10^−2^ (Rayleigh damping) was introduced to model the effect of dissipation of the liquid metal and the elastomer of the microfluidic channel. The viscoelastic constitutive model with a Prony series fitted with experimental results (dynamic mechanical analysis) was adopted by the silicone elastomer of the membrane. The density (*ρ*) of materials were *ρ*_metal_ = 6.25 g/cm^3^ for the liquid metal, *ρ* = 1.07 g/cm^3^ for the channel walls, and *ρ*_membrane_ = 1.07 g/cm^3^ for the membrane, respectively.

### 3D Digital image correlation

Advanced 3D imaging technique, 3D-DIC, was used in this work as a critical tool to quantify surface deformation and compare FEA and experimental results^57^. 3D-DIC experiments used two high-speed cameras (2048 × 1088 in resolution; HT-2000M, Emergent) with 35-mm imaging lenses (F1.4 manual focus; Kowa). Supplementary Fig. 7a presents the schematics of the programmable surface in a magnetic field integrated with 3D imaging capabilities. To minimize errors associated with the ultrathin and transparent programmable membrane, both extrinsic and intrinsic camera parameters were optimized including f-number, illumination, focal length, distortion, light sensitivity, and gain systems. Images of multiple checkerboard patterns^71^ served as the basis for correcting image distortions as shown in Supplementary Fig. 7b. The overall mean reprojection error after the correction was <0.5 pixels for all cameras (Supplementary Fig. 7c). The programmable surfaces were uniformly coated with black dots (~200–500 μm) using a spray-painting technique^72^. The 3D imaging acquisition hardware and the programmable surface system were externally synchronized to allow rapid, automated collection of data for thousands of different shapes. The investigation volume was 40 × 40 × 10 mm^3^, and RSEM between true calibration and 3D reconstructed points was ~70 μm (Supplementary Fig. 7d). 3D reconstruction error statistics show that the median values of difference between true calibration points and 3D reconstructed points were ~15, 15, and 7 µm for *x*, *y*, and *z* coordinates, respectively. To achieve high resolution and accurate deformation characteristics, the DIC subset radius and spacing were set as 30 and 15 pixels, resolving over 1500 grids. Data points with high correlation coefficients (>0.5 max correlation coefficient), in which indicated inconsistencies between two image sets due to the light reflection or/and transparency of programmable surfaces, were filtered out to minimize errors during the 3D reconstruction process. Filtered datasets were grid-interpolated into 20 by 20 grid points to generate consistent data points and grid locations across all set of experiments.

## Supplementary information

Supplementary Information

Description of Additional Supplementary Files

Supplementary Movie 1

Supplementary Movie 2

Supplementary Movie 3

Supplementary Movie 4

Supplementary Movie 5

Supplementary Movie 6

Supplementary Movie 7

Supplementary Movie 8

Supplementary Movie 9

Supplementary Movie 10

## Data Availability

The data that support the findings of this study are available from the corresponding authors upon reasonable request.

## Code availability

The codes that support the findings of this study are available from the corresponding authors upon reasonable request.

## References

[1] Chen T, Bilal OR, Lang R, Daraio C, Shea K (2019). Autonomous deployment of a solar panel using elastic origami and distributed shape-memory-polymer actuators. Phys. Rev. Appl..

[2] Zareei A, Deng B, Bertoldi K (2020). Harnessing transition waves to realize deployable structures. Proc. Natl Acad. Sci. U.S.A..

[3] Kim Y, Yuk H, Zhao R, Chester SA, Zhao X (2018). Printing ferromagnetic domains for untethered fast-transforming soft materials. Nature.

[4] Kotikian A (2019). Untethered soft robotic matter with passive control of shape morphing and propulsion. Sci. Robot..

[5] Zhang F (2021). Rapidly deployable and morphable 3D mesostructures with applications in multimodal biomedical devices. Proc. Natl Acad. Sci. U.S.A..

[6] Lind JU (2017). Instrumented cardiac microphysiological devices via multimaterial three-dimensional printing. Nat. Mater..

[7] Yao S (2020). Nanomaterial‐enabled flexible and stretchable sensing systems: processing, integration, and applications. Adv. Mater..

[8] Bolaños Quiñones VA, Zhu H, Solovev AA, Mei Y, Gracias DH (2018). Origami biosystems: 3D assembly methods for biomedical applications. Adv. Biosyst..

[9] Park S (2021). Adaptive and multifunctional hydrogel hybrid probes for long-term sensing and modulation of neural activity. Nat. Commun..

[10] Ballew C, Roberts G, Camayd-Muñoz S, Debbas MF, Faraon A (2021). Mechanically reconfigurable multi-functional meta-optics studied at microwave frequencies. Sci. Rep..

[11] Spägele C (2021). Multifunctional wide-angle optics and lasing based on supercell metasurfaces. Nat. Commun..

[12] Ding M (2020). Multifunctional soft machines based on stimuli-responsive hydrogels: from freestanding hydrogels to smart integrated systems. Mater. Today Adv..

[13] Pikul JH (2017). Stretchable surfaces with programmable 3D texture morphing for synthetic camouflaging skins. Science.

[14] Terwagne D, Brojan M, Reis PM (2014). Smart morphable surfaces for aerodynamic drag control. Adv. Mater..

[15] Tuo Y (2019). Drag reduction of anisotropic superhydrophobic surfaces prepared by laser etching. Langmuir.

[16] Liu K, Hacker F, Daraio C (2021). Robotic surfaces with reversible, spatiotemporal control for shape morphing and object manipulation. Sci. Robot..

[17] Cheng Y, Lu H, Lee X, Zeng H, Priimagi A (2020). Kirigami-based light-induced shape-morphing and locomotion. Adv. Mater..

[18] Zeng H (2015). Light-fueled microscopic walkers. Adv. Mater..

[19] Mohd Jani J, Leary M, Subic A, Gibson MA (2014). A review of shape memory alloy research, applications and opportunities. Mater. Des..

[20] Rodriguez, J. N. et al. Shape-morphing composites with designed micro-architectures. *Sci. Rep*. **6**, 27933 (2016). 10.1038/srep27933 PMC4908431 27301435

[21] Jiang Z (2020). Strong, self-healable, and recyclable visible-light‐responsive hydrogel actuators. Angew. Chem..

[22] Kim H (2019). Light‐driven shape morphing, assembly, and motion of nanocomposite gel surfers. Adv. Mater..

[23] Sydney Gladman A, Matsumoto EA, Nuzzo RG, Mahadevan L, Lewis JA (2016). Biomimetic 4D printing. Nat. Mater..

[24] Jager EWH (2000). Microrobots for micrometer-size objects in aqueous media: potential tools for single-cell manipulation. Science.

[25] Hajiesmaili E, Clarke DR (2019). Reconfigurable shape-morphing dielectric elastomers using spatially varying electric fields. Nat. Commun..

[26] Wang J, Li S, Gao D, Xiong J, Lee PS (2019). Reconfigurable and programmable origami dielectric elastomer actuators with 3D shape morphing and emissive architectures. NPG Asia Mater..

[27] Lee H (2019). Directional shape morphing transparent walking soft robot. Soft Robot..

[28] Kim H (2018). Biomimetic color changing anisotropic soft actuators with integrated metal nanowire percolation network transparent heaters for soft robotics. Adv. Funct. Mater..

[29] Wang S (2020). Asymmetric elastoplasticity of stacked graphene assembly actualizes programmable untethered soft robotics. Nat. Commun..

[30] Zhang X (2014). Photoactuators and motors based on carbon nanotubes with selective chirality distributions. Nat. Commun..

[31] Xu S (2015). Assembly of micro/nanomaterials into complex, three-dimensional architectures by compressive buckling. Science.

[32] Zhang, Y. et al. Printing, folding and assembly methods for forming 3D mesostructures in advanced materials. *Nat. Rev. Mater*. **2**, 17019 (2017).

[33] Luan, H. et al. Complex 3D microfluidic architectures formed by mechanically guided compressive buckling. *Sci. Adv*. **7**, eabj3686 (2021). 10.1126/sciadv.abj3686 PMC8528415 34669471

[34] An N, Domel AG, Zhou J, Rafsanjani A, Bertoldi K (2020). Programmable hierarchical Kirigami. Adv. Funct. Mater..

[35] Lin G (2017). Cuts guided deterministic buckling in arrays of soft parallel plates for multifunctionality. ACS Appl. Mater. Interfaces.

[36] Dieleman P, Vasmel N, Waitukaitis S, van Hecke M (2020). Jigsaw puzzle design of pluripotent origami. Nat. Phys..

[37] Miskin MZ (2018). Graphene-based bimorphs for micron-sized, tautonomous origami machines. Proc. Natl Acad. Sci. U.S.A..

[38] Huang Q (2020). Solvent responsive self‐folding of 3D photosensitive graphene architectures. Adv. Intell. Syst..

[39] Xu W (2019). Self-folding hybrid graphene skin for 3D biosensing. Nano Lett..

[40] Boley JW (2019). Shape-shifting structured lattices via multimaterial 4D printing. Proc. Natl Acad. Sci. U.S.A..

[41] McMahan C (2018). Shape-morphing architected sheets with non-periodic cut patterns. Soft Matter.

[42] Ni X (2019). 2D mechanical metamaterials with widely tunable unusual modes of thermal expansion. Adv. Mater..

[43] Weng S (2021). 4D printing of glass fiber-regulated shape shifting structures with high stiffness. ACS Appl. Mater. Interfaces.

[44] Jin B (2018). Programming a crystalline shape memory polymer network with thermo- and photo-reversible bonds toward a single-component soft robot. Sci. Adv..

[45] Nojoomi A, Arslan H, Lee K, Yum K (2018). Bioinspired 3D structures with programmable morphologies and motions. Nat. Commun..

[46] Zhao X (2011). Active scaffolds for on-demand drug and cell delivery. Proc. Natl Acad. Sci. U.S.A..

[47] Melancon D, Gorissen B, García-Mora CJ, Hoberman C, Bertoldi K (2021). Multistable inflatable origami structures at the metre scale. Nature.

[48] Mao G (2020). Soft electromagnetic actuators. Sci. Adv..

[49] Ford MJ (2019). A multifunctional shape-morphing elastomer with liquid metal inclusions. Proc. Natl Acad. Sci. U.S.A..

[50] Haines CS (2014). Artificial muscles from fishing line and sewing thread. Science.

[51] Na J-H (2015). Programming reversibly self-folding origami with micropatterned photo-crosslinkable polymer trilayers. Adv. Mater..

[52] Zhang H, Guo X, Wu J, Fang D, Zhang Y (2018). Soft mechanical metamaterials with unusual swelling behavior and tunable stress-strain curves. Sci. Adv..

[53] Ma C (2014). Supramolecular lego assembly towards three-dimensional multi-responsive hydrogels. Adv. Mater..

[54] Fu H (2018). Morphable 3D mesostructures and microelectronic devices by multistable buckling mechanics. Nat. Mater..

[55] Yan Z (2016). Mechanical assembly of complex, 3D mesostructures from releasable multilayers of advanced materials. Sci. Adv..

[56] Bai, Y. et al. A dynamically reprogrammable metasurface with self-evolving shape morphing. arXiv:2112.04631. https://arxiv.org/abs/2112.04631 (2021).

[57] Solav D, Moerman KM, Jaeger AM, Genovese K, Herr HM (2018). MultiDIC: an open-source toolbox for multi-view 3D digital image correlation. IEEE Access.

[58] Fu, H., Bai, K., Huang, Y. & Zhang, Y. Recent progress of morphable 3D mesostructures in advanced materials. *J. Semicond*. **41**, 041604 (2020).

[59] Won, P. et al. Transparent soft actuators/sensors and camouflage skins for imperceptible soft robotics. *Adv. Mater*. **33**, 2002397 (2021). 10.1002/adma.202002397 33089569

[60] Kim H (2020). Shape morphing smart 3D actuator materials for micro soft robot. Mater. Today.

[61] Briggs LJ (1957). Gallium: thermal conductivity; supercooling; negative pressure. J. Chem. Phys..

[62] Kuder IK, Arrieta AF, Raither WE, Ermanni P (2013). Variable stiffness material and structural concepts for morphing applications. Prog. Aerosp. Sci..

[63] Wang W, Ahn S-H (2017). Shape memory alloy-based soft gripper with variable stiffness for compliant and effective grasping. Soft Robot.

[64] Imamura H, Kadooka K, Taya M (2017). A variable stiffness dielectric elastomer actuator based on electrostatic chucking. Soft Matter.

[65] Wang Y, Li L, Hofmann D, Andrade JE, Daraio C (2021). Structured fabrics with tunable mechanical properties. Nature.

[66] Chen T, Pauly M, Reis PM (2021). A reprogrammable mechanical metamaterial with stable memory. Nature.

[67] Di Lillo L, Raither W, Bergamini A, Zündel M, Ermanni P (2013). Tuning the mechanical behaviour of structural elements by electric fields. Appl. Phys. Lett..

[68] Murray G, Gandhi F (2010). Multi-layered controllable stiffness beams for morphing: energy, actuation force, and material strain considerations. Smart Mater. Struct..

[69] Raither W, Heymanns M, Bergamini A, Ermanni P (2013). Morphing wing structure with controllable twist based on adaptive bending–twist coupling. Smart Mater. Struct..

[70] Camacho JM, Sosa V (2013). Alternative method to calculate the magnetic field of permanent magnets with azimuthal symmetry. Rev. Mex. Fisica Sci. E.

[71] Zhang Z (2000). A flexible new technique for camera calibration. IEEE Trans. Pattern Anal. Mach. Intell..

[72] Liu C (2021). Wireless, skin‐interfaced devices for pediatric critical care: application to continuous, noninvasive blood pressure monitoring. Adv. Healthc. Mater..

