# Supplementary material for: Soft shape-programmable surfaces by fast electromagnetic actuation of liquid metal networks
Source: Nat Commun. 2022 Sep 23;13:5576. doi: 10.1038/s41467-022-31092-y (PMC9508113; doi:10.1038/s41467-022-31092-y)
Supplement: Supplementary file 1 — Supplementary Information [file 41467_2022_31092_MOESM1_ESM.pdf]

Supplementary Information

Soft shape-programmable surfaces by fast electromagnetic actuation of liquid metal networks

Supplementary Note

The target shapes are defined via the superimposition of several Gaussian functions, in the following form

$$\frac{z}{L}=\sum_{i=1}^{8}c_i\exp\left[-\frac{10\left(x-x_i\right)^2+10\left(y-y_i\right)^2}{L^2}\right],$$

with z denoting the out-of-plane coordinate, x and y denoting the in-plane coordinates. Values of x_i and y_i are listed in Supplementary Table 2. The six target shapes presented in Fig. 3 (shapes 1, 2, 3 in Fig. 3a and shapes 4, 5, 6 in Fig. 3b, ordered from top to bottom in each sub figure) are defined by taking c_i as listed in Supplementary Table 3, followed by normalizing each shape such that the maximum out-of-plane displacement is 3 mm.

Supplementary Figures

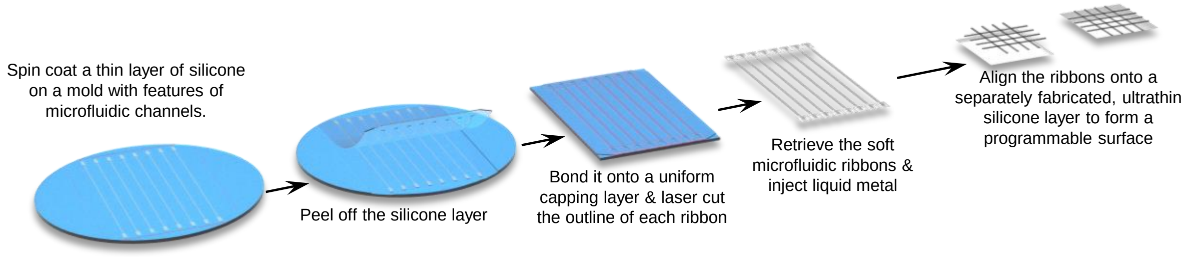

Supplementary Fig. 1 Schematic illustration of the process to fabricate the programmable surface based on liquid metal microfluidics.

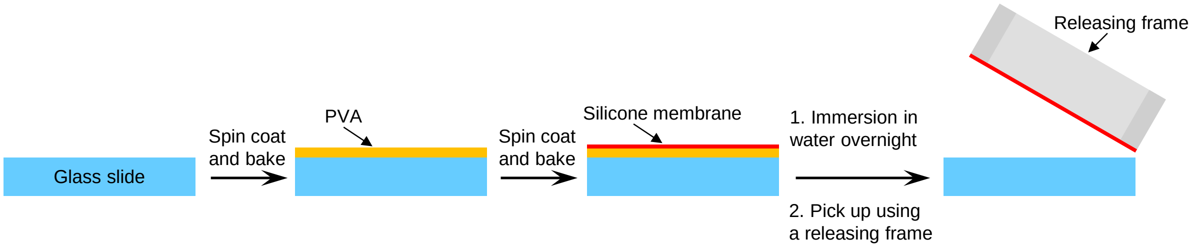

Supplementary Fig. 2 Schematic illustration of the process to fabricate the ultrathin freestanding silicone membrane.

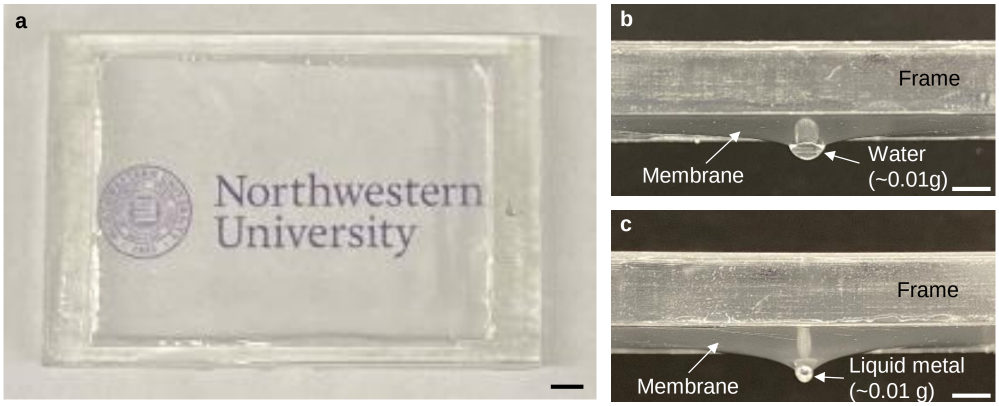

Supplementary Fig. 3 Freestanding ultrathin silicone membrane. a Top-down view of a transparent ultrathin (~5 µm) silicone membrane. **b, c** Side view of the ultrathin silicone membrane deforming under the weight of (**b**) water and (**c**) liquid metal droplets (~0.01 g), respectively. Scale bars, 0.5 mm.

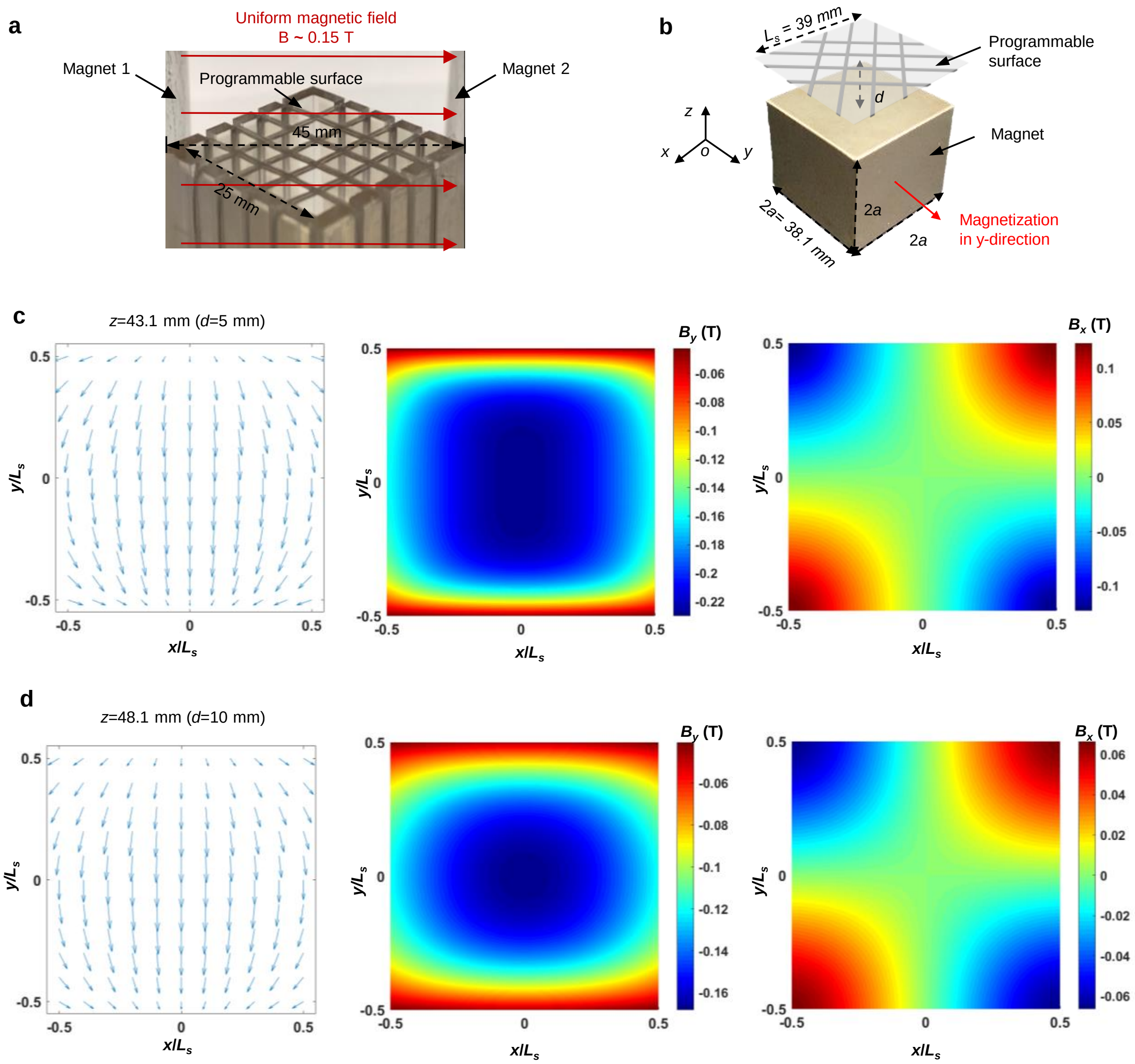

Supplementary Fig. 4 Distribution of magnetic fields. **a** Schematic illustration of a uniform magnetic field associated with two permanent disk magnets (diameter = 76.2 mm, thickness = 12.7 mm) placed in parallel and the position of the programmable surface. **b—d** Non-uniform magnetic field. **b** Schematic illustration of a non-uniform magnetic field associated with a permanent cuboidal block magnet and the position of the programmable surface. **c, d** Distribution of the non-uniform magnetic field in the *xy* plane at a distance of 5 mm (**c**) and 10 mm (**d**) above the top surface of the magnet, respectively. Left panel: direction of magnetic field; middle panel: magnetic field strength in *y*-direction; right panel: magnetic field strength in *x*-direction.

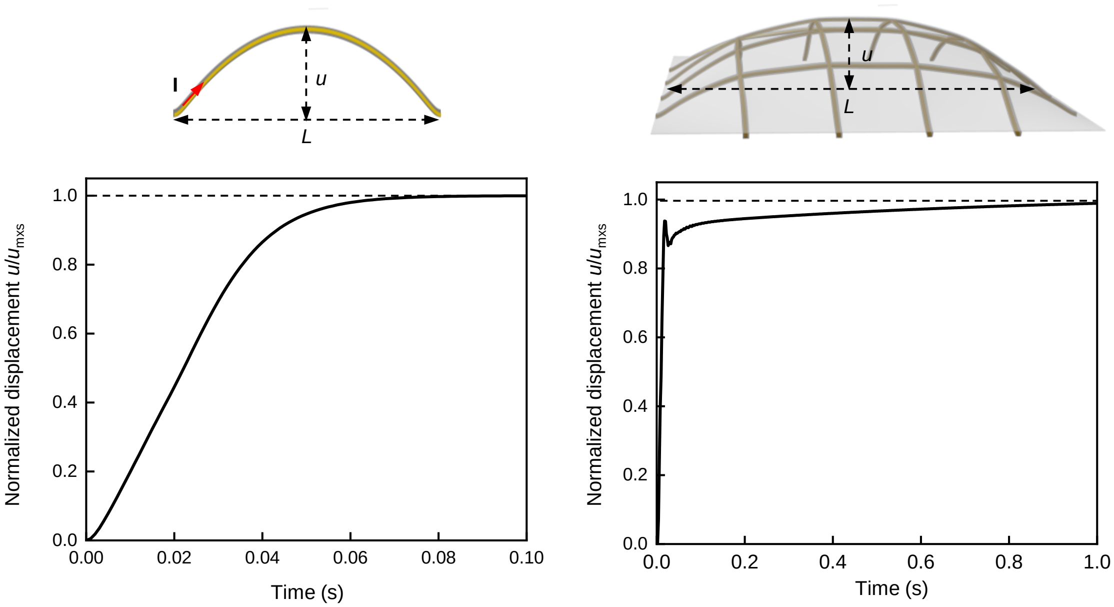

Supplementary Fig. 5 FEA results for viscoelastic responses. Plots of the normalized displacement (u/u_{max}, u_{max} = 4 mm) as a function of time after the structures are actuated. Left: an isolated ribbon; right: a surface incorporating a 4-by-4 array of ribbons.

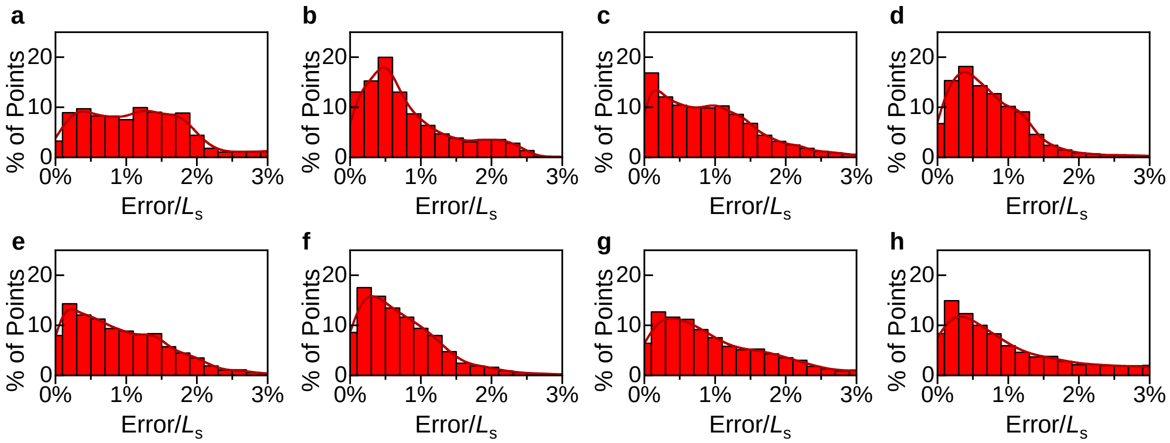

Supplementary Fig. 6 Quantitative comparison via error analysis. a—d Histogram plots of errors (normalized by the surface length, L_s) between FEA predictions and 3D Digital Image Correlation (3D-DIC) results for shapes I—IV, respectively as presented in Fig. 2a. **e—f** Histogram plots of errors (normalized by the surface length, L_s) between FEA predictions and 3D-DIC results for shapes I—IV, respectively, as presented in Fig. 2b.

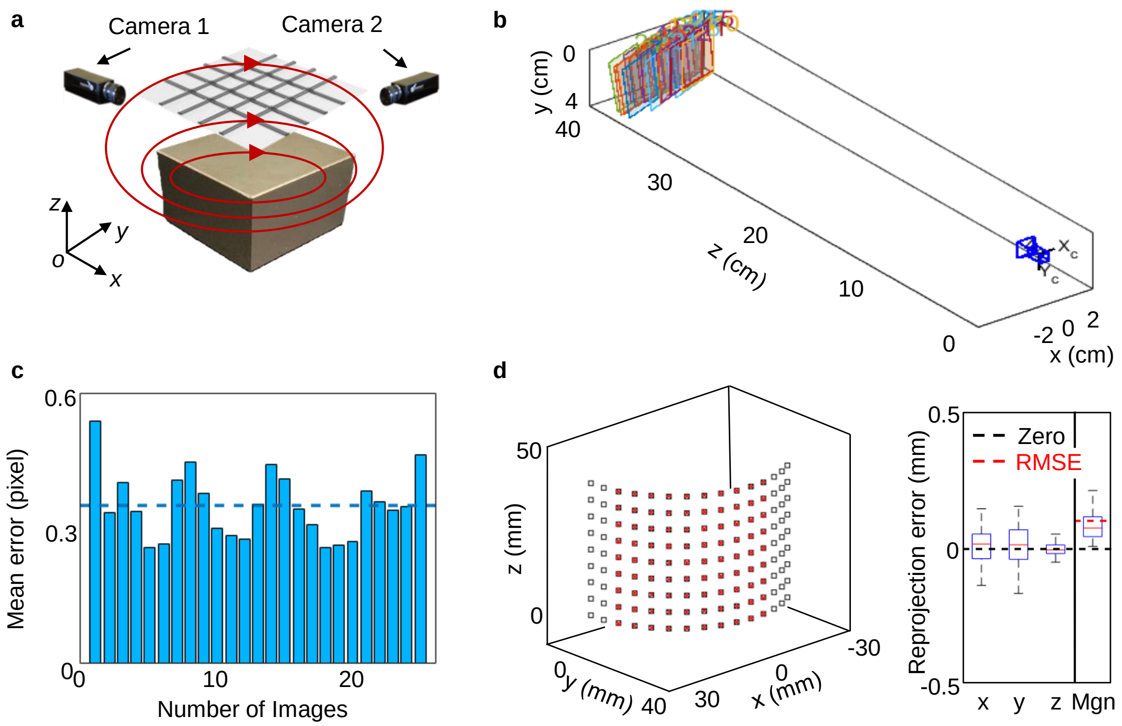

Supplementary Fig. 7 3D-DIC setup and error analysis. a Schematics of the 3D-DIC system used to quantify 3D surface deformation. **b** Extrinsic parameters visualization for camera 1: camera location (blue camera symbol) with respect to 25 images of calibration patterns (different colored planes). **c** Mean projection error per image for camera 1. The dashed blue line denotes the overall mean reprojection error after the distortion correction. **d** Left: True calibration points (black squares) vs. reconstructed points (red crosses); right: 3D reconstruction error statistics.

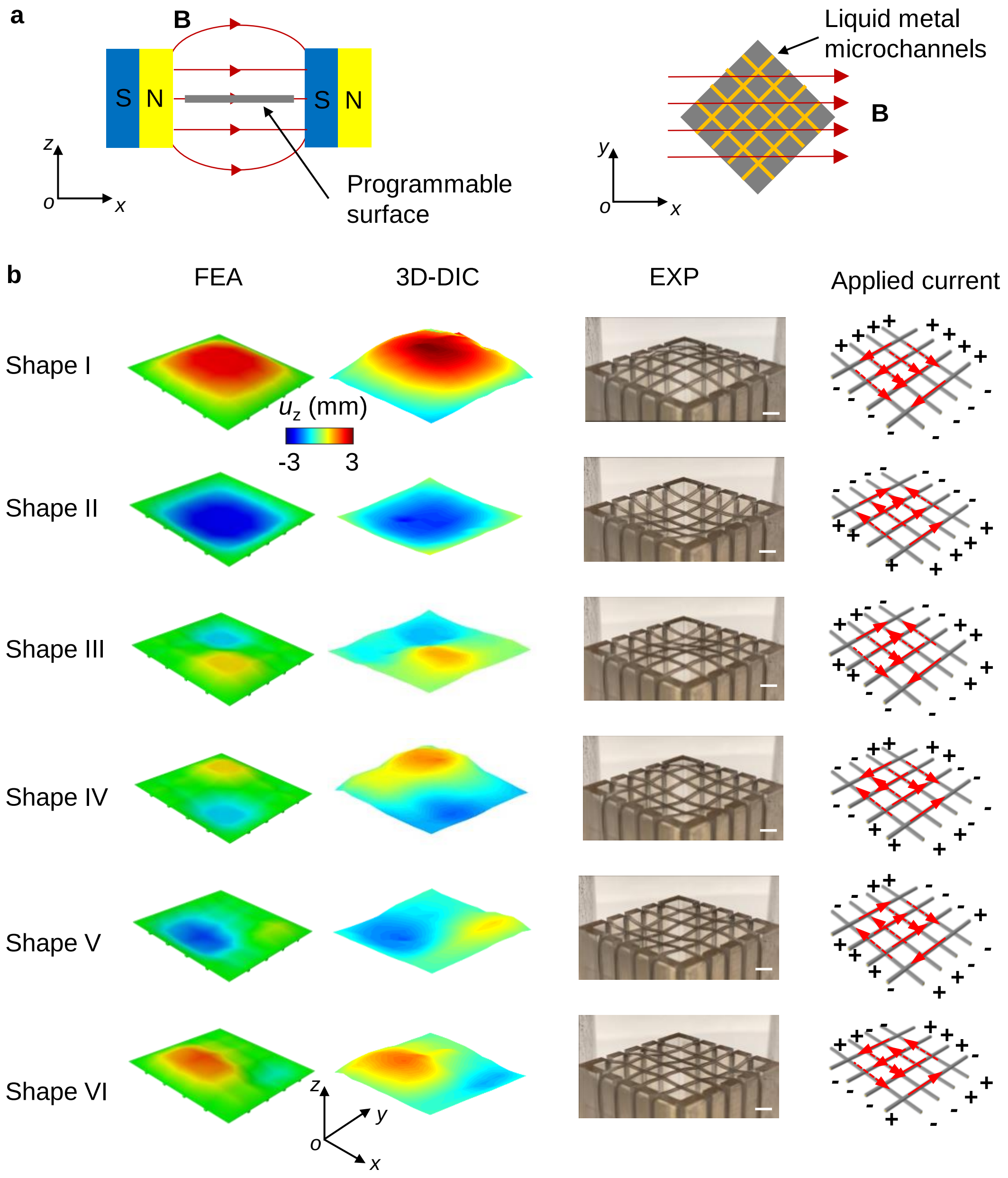

Supplementary Fig. 8 Programmable surface in a uniform magnetic field. **a** Schematic illustration of the programmable surface in a uniform magnetic field created by two disk magnets in parallel, as presented in Supplementary Fig. 4. **b** FEA predictions and experimental results (3D-DIC and optical) of a set of shapes realized by the programmable surface in the uniform magnetic field. Scale bars, 500 µm.

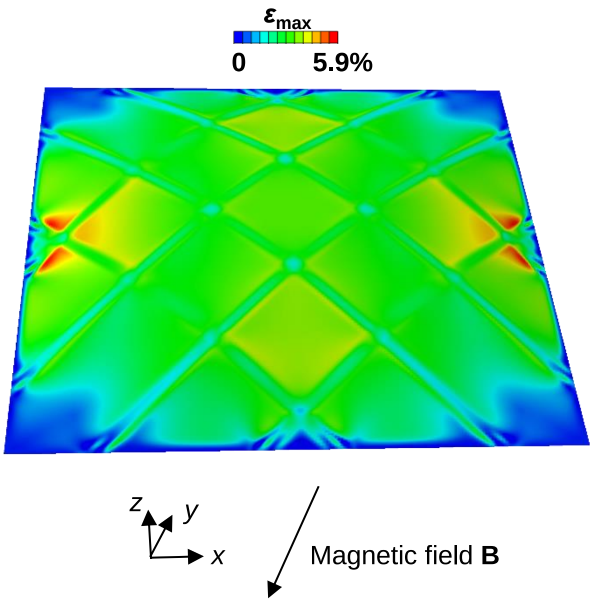

Supplementary Fig. 9 FEA results for the principal strain distribution in the membrane for shape I in Fig. 2a.

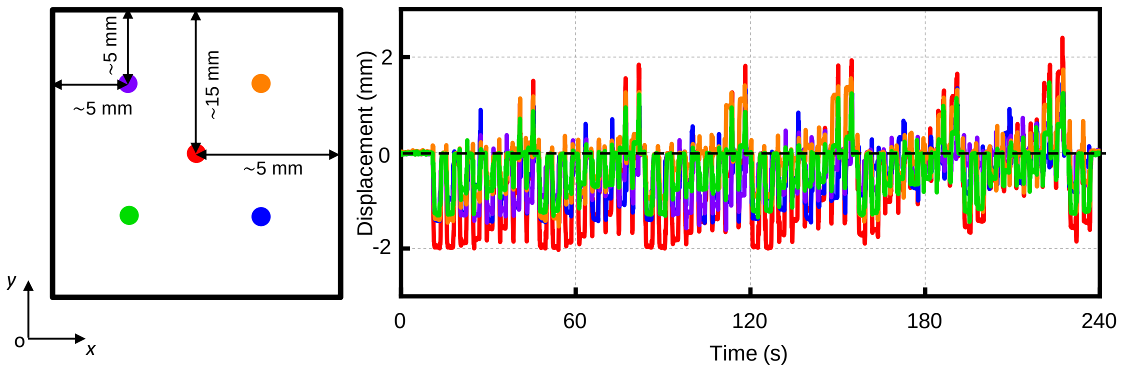

Supplementary Fig. 10 Displacements of five representative points on the surface (left) as a function of time as the programmable surface goes through 50 consecutive shape transformations and returns to the initial 2D planar configuration between each transformation.

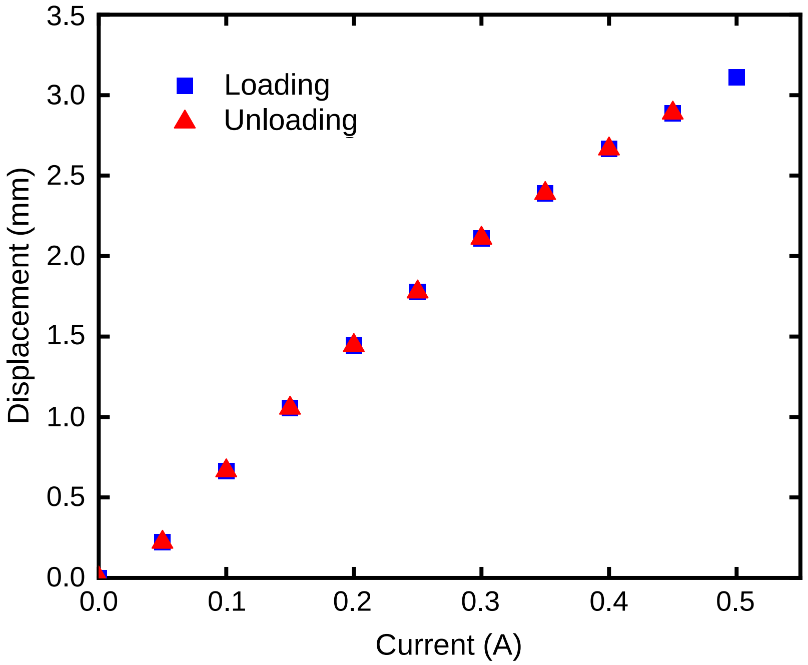

Supplementary Fig. 11 Displacement response of the center point on the programmable surface through a cycle of loading and unloading associated with the application and removal of applied current.

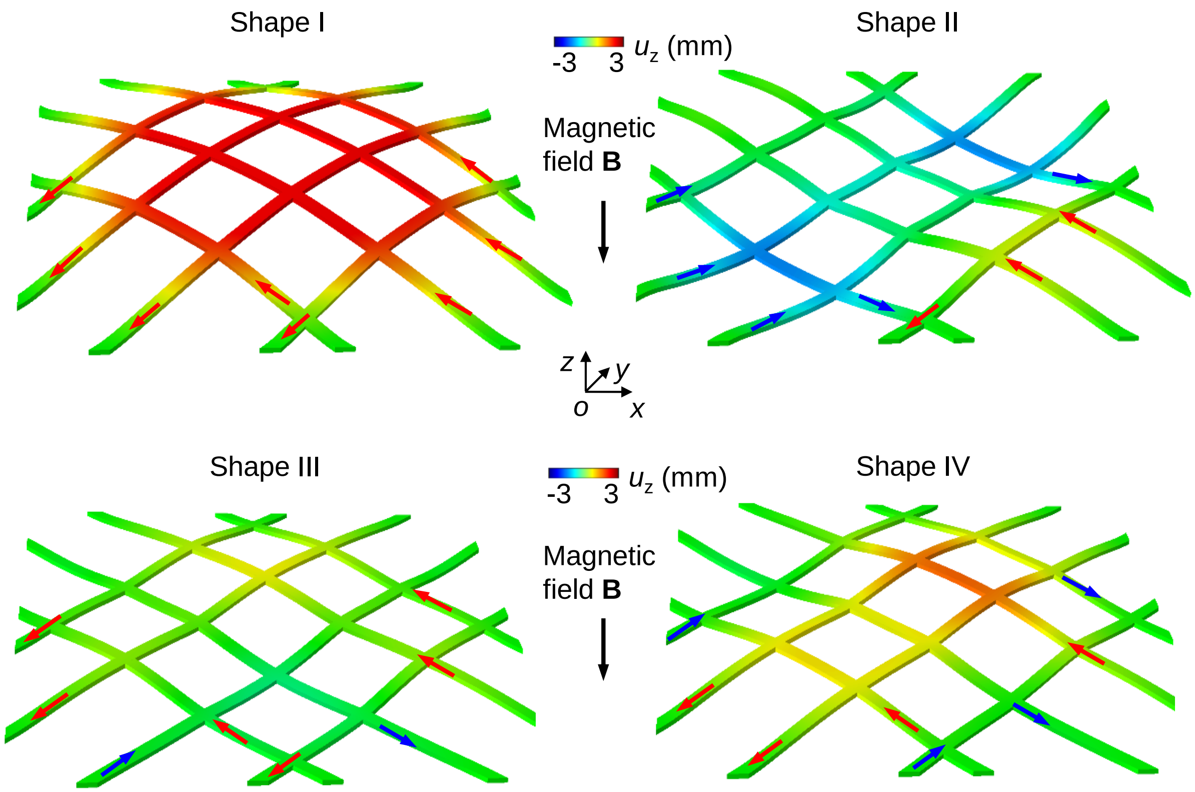

Supplementary Fig. 12 FEA illustration of the deformations of the underlying liquid metal ribbons and the applied currents that correspond to the 3D shapes shown in Fig. 2a. The arrows indicate the current directions, with red arrows denoting the current direction that generates Lorentz force along the positive z-direction and blue arrows denoting the current direction that generates Lorentz force along the negative z-direction.

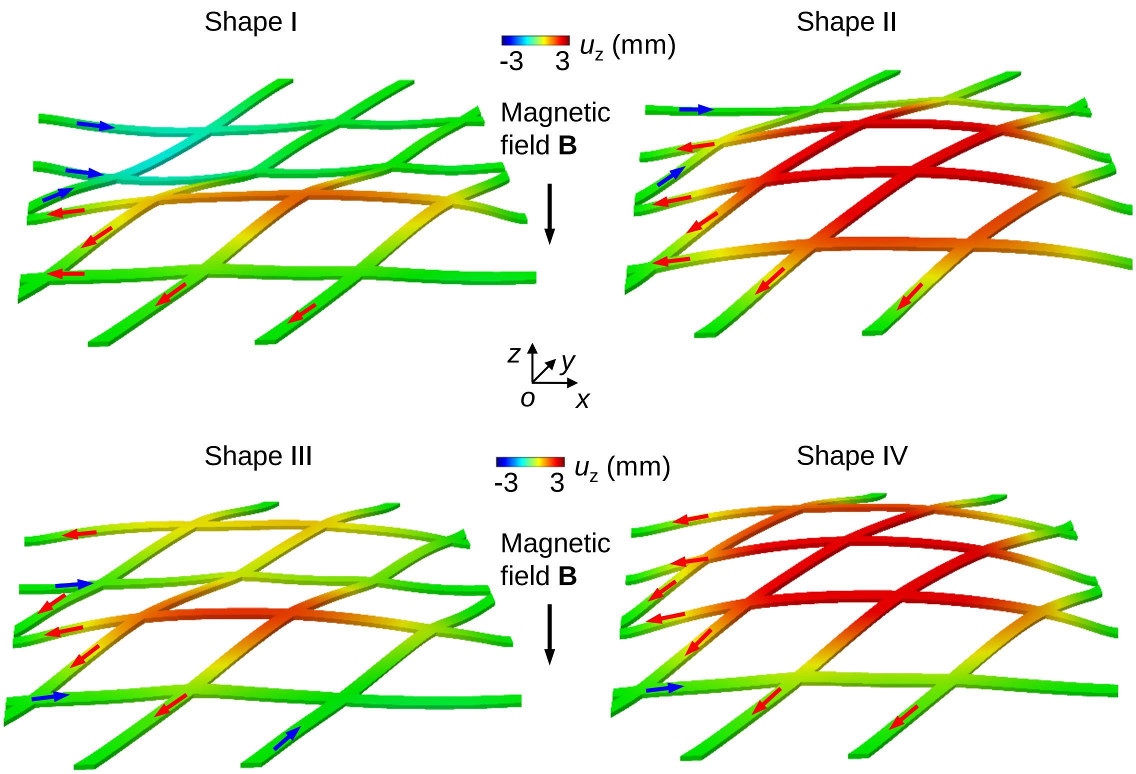

Supplementary Fig. 13 FEA illustration of the deformations of the underlying liquid metal ribbons and the applied currents that correspond to the 3D shapes shown in Fig. 2b. The arrows indicate the current directions, with red arrows denoting the current direction that generates Lorentz force along the positive *z*-direction and blue arrows denoting the current direction that generates Lorentz force along the negative *z*-direction.

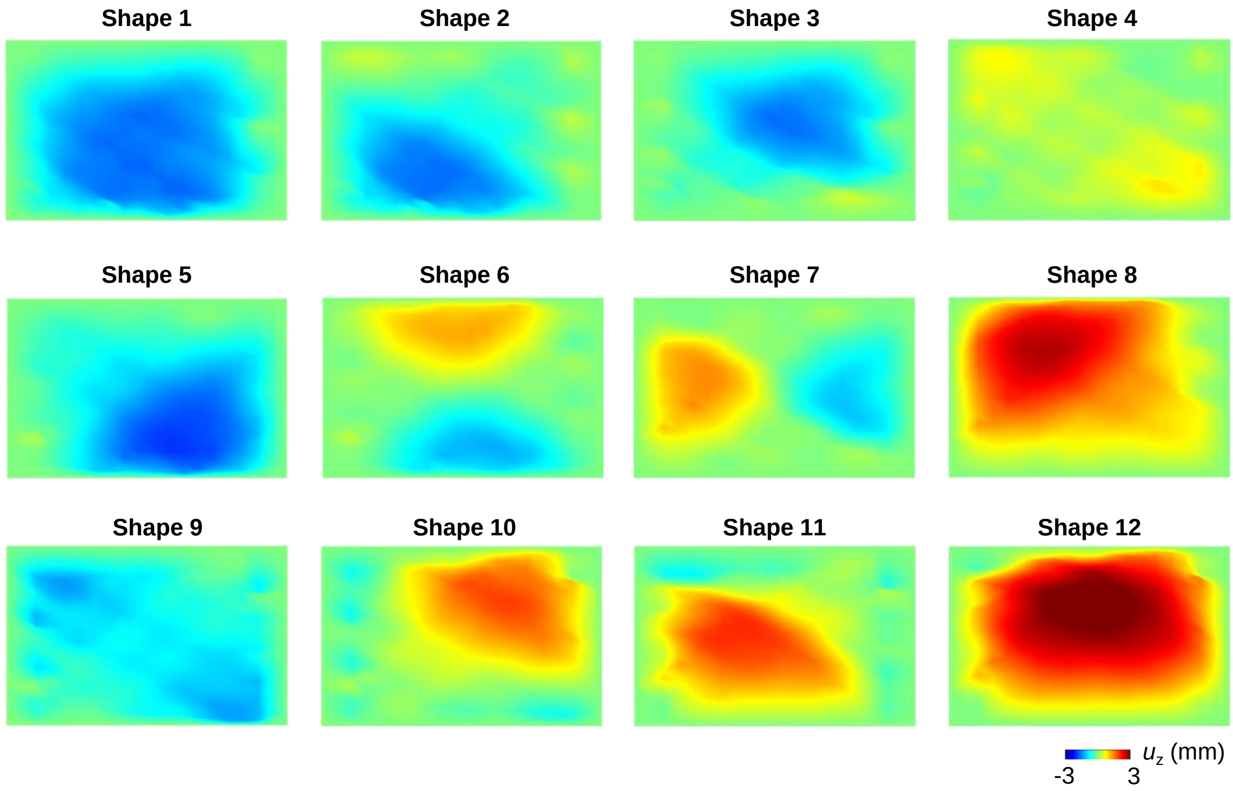

Supplementary Fig. 14 A collection of representative shapes (3D-DIC images) from the shape library of the programmable surface with a 45º/-45º ribbon layout.

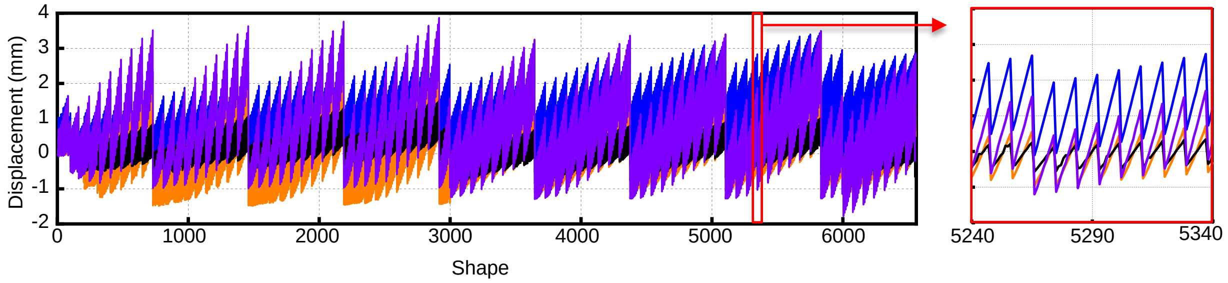

Supplementary Fig. 15 Displacements of eight representative points of the ~7000 shapes in the shape library of the programmable surface with a 45º/-45º ribbon layout.

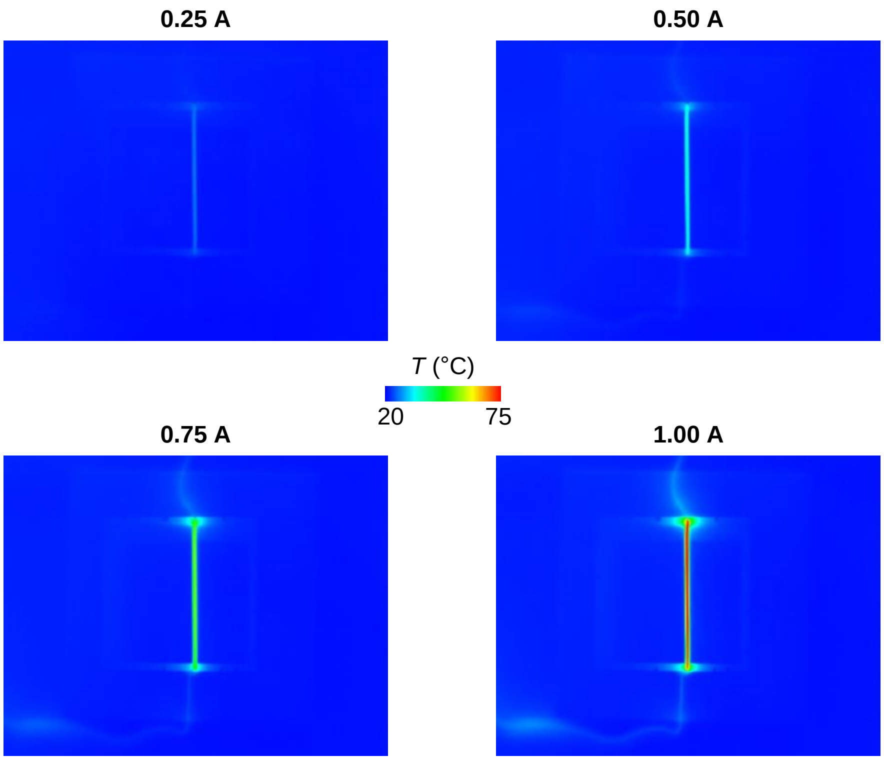

Supplementary Fig. 16 Effect of Joule heating. IR images of an isolated liquid metal ribbon 60 seconds after currents at different intensities are applied.

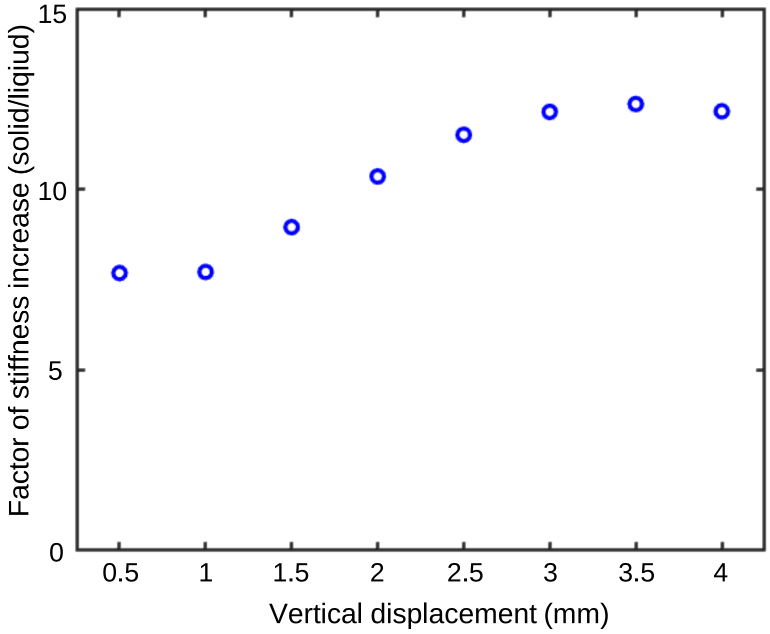

Supplementary Fig. 17 Stiffness increase of the programmable surface due to the solidification of liquid gallium at different compressive vertical displacements.

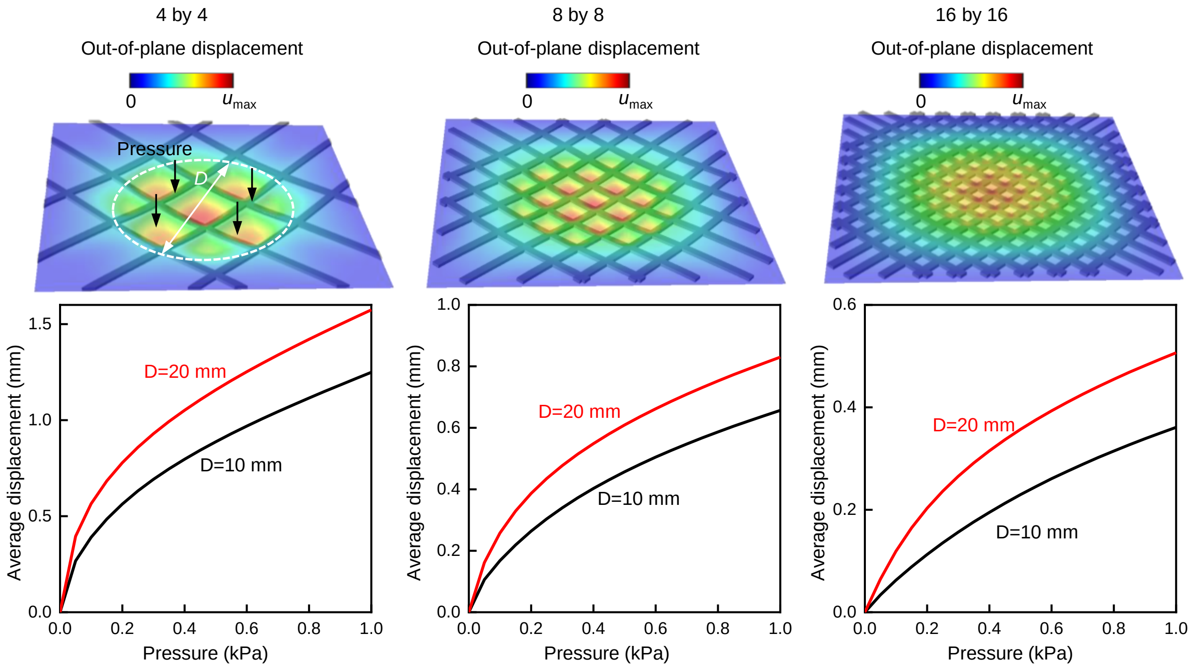

Supplementary Fig. 18 FEA results of deformation of programmable surfaces ($h_{membrane}$ = 100 µm and $E_{membrane}$ = 100 kPa) with different ribbon numbers (4-by-4, 8-by-8, and 16-by-16) in flat, shape-fixed forms (gallium as a solid) under pressure applied in a circular geometry in the center.

Supplementary Tables

Supplementary Table 1 Summary of shape-morphing systems and their response times.

Time scale	Example	Mechanisms/materials	Response time
<1 s	*Adv. Mater.* **27**, 3883–3887 (2015)	Light responsive liquid crystal elastomer	10 ms
	This work	Electromagnetic actuation of liquid metal sealed in elastomeric matrices	50 ms
	Nature **558**, 274–279 (2018)	3D printed silicone composites with magnetic particles	~500 ms
1 s ~ 1 min	*Nat. Commun.* **10**, 10–16 (2019)	Dielectric elastomer actuator	<1 s
	Science **358**, 210–214 (2017)	Pneumatic actuation of silicone elastomer	~5 s
	Science **347**, 154–159 (2015)	Compressive buckling	~20s
	Proc. Natl. Acad. Sci. U. S. A. **116**, 21438–21444 (2019)	Joule heating of liquid metal composite	~30 s
> 1 min	*Sci. Robot.* **6**, eabf5116 (2021)	Heat responsive liquid crystal elastomer	~60 s
	Sci. Adv. **4**, eaar8535 (2018)	Swelling of hydrogel	>15 min

Supplementary Table 2 Values for x_i and y_i.

i	x_i/L	y_i/L	i	x_i/L	y_i/L
1	-0.25	-0.25	5	0	0
2	0	-0.25	6	0.25	0
3	0.25	-0.25	7	-0.25	0.25
4	-0.25	0	8	0	0.25

Supplementary Table 3 Values for c_i.

	c_1	c_2	c_3	c_4	c_5	c_6	c_7	c_8
Shape 1	0	0	0	0	1	0	0	0
Shape 2	1	1	0	1	0	0	0	0
Shape 3	0	-1	0	0	0	0	0	1
Shape 4	0	1	1	0	0	0	-1	-1
Shape 5	0	0	0	1	1	1	0	0
Shape 6	0	1	0	0	-1	0	0	1
