# Supplementary material for: Soft shape-programmable surfaces by fast electromagnetic actuation of liquid metal networks
Source: Nat Commun. 2022 Sep 23;13:5576. doi: 10.1038/s41467-022-31092-y (PMC9508113; doi:10.1038/s41467-022-31092-y)
Supplement: Supplementary file 2 — Description of Additional Supplementary Files [file 41467_2022_31092_MOESM2_ESM.docx]

**Supplementary Movie Legends**

**Supplementary Movie 1 Full shape development and shape switching in slow motion.**

**Supplementary Movie 2 Shape transformations between 20 different shapes of a programmable surface with a 45°/-45° ribbon design as presented in Fig. 2a.**

**Supplementary Movie 3 Shape transformations between 20 different shapes of a programmable surface with a 0°/45° ribbon design as presented in Fig. 2b.**

**Supplementary Movie 4 Shape transformations between target shapes of a programmable surface with a 45°/-45° ribbon design as presented in Fig. 3a.**

**Supplementary Movie 5 Shape transformations between target shapes of a programmable surface with a 0°/45° ribbon design as presented in Fig. 3b.**

**Supplementary Movie 6 Shape fixation and reprogramming utilizing liquid metal phase transition as presented in Fig. 3c.**

**Supplementary Movie 7 Load bearing capability as presented in Fig. 3e.**

**Supplementary Movie 8 4D programmability as presented in Fig. 4.**

**Supplementary Movie 9 Noise cancellation capability as presented in Fig. 5.**

**Supplementary Movie 10 A 3D dynamic display as presented in Fig. 6.**
